# Women for science and science for women: Gaps, challenges and opportunities towards optimizing pre-exposure prophylaxis for HIV-1 prevention

**DOI:** 10.3389/fimmu.2022.1055042

**Published:** 2022-12-06

**Authors:** Quarraisha Abdool Karim, Derseree Archary, Françoise Barré-Sinoussi, Kristina Broliden, Cecilia Cabrera, Francesca Chiodi, Sarah J. Fidler, Tanuja N. Gengiah, Carolina Herrera, Ayesha B. M. Kharsany, Lenine J. P. Liebenberg, Sharana Mahomed, Elisabeth Menu, Christiane Moog, Gabriella Scarlatti, Nabila Seddiki, Aida Sivro, Mariangela Cavarelli

**Affiliations:** ^1^ Centre for the AIDS Programme of Research in South Africa (CAPRISA), Doris Duke Medical Research Institute (2Floor), Nelson R Mandela School of Medicine, University of KwaZulu-Natal, Durban, South Africa; ^2^ Department of Epidemiology, Mailman School of Public Health, Columbia University, New York, NY, United States; ^3^ Department of Medical Microbiology, School of Laboratory Medicine and Medical Sciences, University of KwaZulu-Natal, Durban, South Africa; ^4^ Institut Pasteur, Paris, France; ^5^ Department of Medicine Solna, Division of Infectious Diseases, Karolinska Institutet, Department of Infectious Diseases, Karolinska University Hospital, Center for Molecular Medicine, Stockholm, Sweden; ^6^ AIDS Research Institute IrsiCaixa, Institut de Recerca en Ciències de la Salut Germans Trias i Pujol (IGTP), Hospital Germans Trias i Pujol, Universitat Autònoma de Barcelona, Barcelona, Spain; ^7^ Department of Microbiology, Tumor and Cell Biology, Karolinska Institutet, Stockholm, Sweden; ^8^ Department of Infectious Disease, Faculty of Medicine, Imperial College London UK and Imperial College NIHR BRC, London, United Kingdom; ^9^ Department of Infectious Disease, Section of Virology, Faculty of Medicine, Imperial College London, London, United Kingdom; ^10^ Université Paris-Saclay, Inserm, CEA, Center for Immunology of Viral, Auto-immune, Hematological and Bacterial diseases (IMVA-HB/IDMIT), Fontenay-aux-Roses & Le Kremlin-Bicêtre, France; ^11^ MISTIC Group, Department of Virology, Institut Pasteur, Paris, France; ^12^ Laboratoire d’ImmunoRhumatologie Moléculaire, Institut national de la santé et de la recherche médicale (INSERM) UMR_S 1109, Institut thématique interdisciplinaire (ITI) de Médecine de Précision de Strasbourg, Transplantex NG, Faculté de Médecine, Fédération Hospitalo-Universitaire OMICARE, Fédération de Médecine Translationnelle de Strasbourg (FMTS), Université de Strasbourg, Strasbourg, France; ^13^ Viral Evolution and Transmission Unit, IRCCS Ospedale San Raffaele, Milan, Italy; ^14^ JC Wilt Infectious Disease Research Centre, National Microbiology Laboratory, Public Health Agency of Canada, Winnipeg, MB, Canada

**Keywords:** HIV, women, prevention, gender equity, PrEP, mucosal transmission, inflammation, microbiome

## Abstract

Preventing new HIV infections remains a global challenge. Young women continue to bear a disproportionate burden of infection. Oral pre-exposure prophylaxis (PrEP), offers a novel women-initiated prevention technology and PrEP trials completed to date underscore the importance of their inclusion early in trials evaluating new HIV PrEP technologies. Data from completed topical and systemic PrEP trials highlight the role of gender specific physiological and social factors that impact PrEP uptake, adherence and efficacy. Here we review the past and current developments of HIV-1 prevention options for women with special focus on PrEP considering the diverse factors that can impact PrEP efficacy. Furthermore, we highlight the importance of inclusion of female scientists, clinicians, and community advocates in scientific efforts to further improve HIV prevention strategies.

## Introduction

In 2021, among the estimated 38.4 million people living with HIV-1, 54% were adolescent girls and women (1) and HIV/AIDS remains the third leading cause of death globally for women aged 15 to 49 years (2). In 2021 girls and women accounted for 49% of all new infections globally, a frequency that peaked to 63% in sub-Saharan Africa, where six in seven new HIV infections among adolescents aged 15–19 years were among girls (1). In those settings, girls and young women aged 15 to 24 years are twice as likely to be living with HIV compared to their male peers (1). In South Africa, women acquire HIV infection at a younger age, 5 to 7 years before men and are at four times higher risk (3). South Africa is also among the countries with the highest number of pregnant women living with HIV (over one-third), contributing to the highest number of infants born with HIV (4, 5).

Gender inequalities, including unsafe and non-consenting sex, exacerbate girls’ and women’s physiological vulnerability to HIV together with limited HIV prevention options for women and/or access to health services play an important role in contributing to increased HIV risk. Furthermore, domestic violence against women worldwide greatly intensified during the COVID-19 pandemic (6). This was accompanied by a reduced access to medical care, including HIV treatment and prevention, as well as sexual and reproductive services.

Often, young people are denied the information about their own sexual and reproductive health and rights, with most lacking the knowledge required to protect themselves from HIV. This is further compounded by an individuals’ perception of risk. Most persons susceptible to contract HIV do not perceive themselves at high risk. Women may have limited awareness of their partners sexual behaviors, thereby underestimating their own HIV risk, or may feel shame or fear being stigmatized for disclosing their true HIV risk. Self-assessment of risk is challenging when reflecting on intimate relationships and in settings of intimate partner violence for instance, women might feel uncomfortable to discuss or acknowledge their own risk. The impact of these barriers is strongest in high-HIV prevalence settings, predominantly in eastern and southern Africa (7, 8).

Importantly as girls transition from teenagers to adulthood and undergo substantial psychosocial, cognitive, and emotional changes their vulnerability to HIV is enhanced through multiple complex and diverse structural, social, behavioral, and biological co-factors (9–11). Typically, young women have the least amount of power and their HIV risk intersects with poverty, race and geospatial location with those in low-middle income countries bearing the brunt of HIV infection. Notwithstanding, appropriate information, education, guidance, and support could help young girls transition through this volatile developmental period (9). Risk reduction programs in schools are crucial to promote healthy behaviors and help develop young people’s identities to enhance self-esteem and rational decision making to towards safer adulthood (12). A high proportion of young girls are already sexually active (13), and already have a high prevalence of HIV infection. Sexual debut is associated with high rates of unintended pregnancies, sexually transmitted infections (STIs), increased HIV acquisition rates and often leads to poor school completion rates, and in the longer term poorer health and economic outcomes among young girls (12, 14, 15). Schools and primary care clinics where health services are sought, provide opportunities to establish what proportion of sexual activity is consensual and for increasing co-parenting responsibility particularly in teenage pregnancies.

Although women comprise nearly half of people living with HIV, they continue to be largely under-represented in HIV clinical studies particularly if pregnant or lactating or adolescents thereby creating important data gaps and delays in safety and efficacy data for those at highest risk of acquiring HIV. A systematic literature review conducted in 2016 reported that women represented a median of 19.2% participants in antiretroviral treatment/PrEP studies, 38.1% in prophylactic vaccines studies, and 11.1% in HIV cure strategies studies (16). This is partially due to the fact that a large proportion of HIV clinical trials and cure-related research taking place in resource sufficient countries mainly enroll men who have sex with men (MSM). In these settings enrolling women is difficult not only because MSM represent 80% of the persons living with HIV, but also because of the greater stigma affecting women living with HIV compared to men. In contrast the majority of clinical trial participants in low-middle income countries enroll *cis*-women.

This under-representation of women included in clinical trials generates substantial gaps in our understanding of gender differences in virological, immunological and clinical presentation of HIV infection geo-spatially. Importantly, when efficacious prevention and treatment products become available, they are used globally despite these gaps in knowledge. In addition, the effect of clinical interventions (safety and efficacy) can differ because of sex/gender. Yet, current guidelines for using antiretroviral treatments do not distinguish between men and women, apart from some studies conducted in pregnant and post-partum women, from whom we have even less information available.

**Figure 1:**
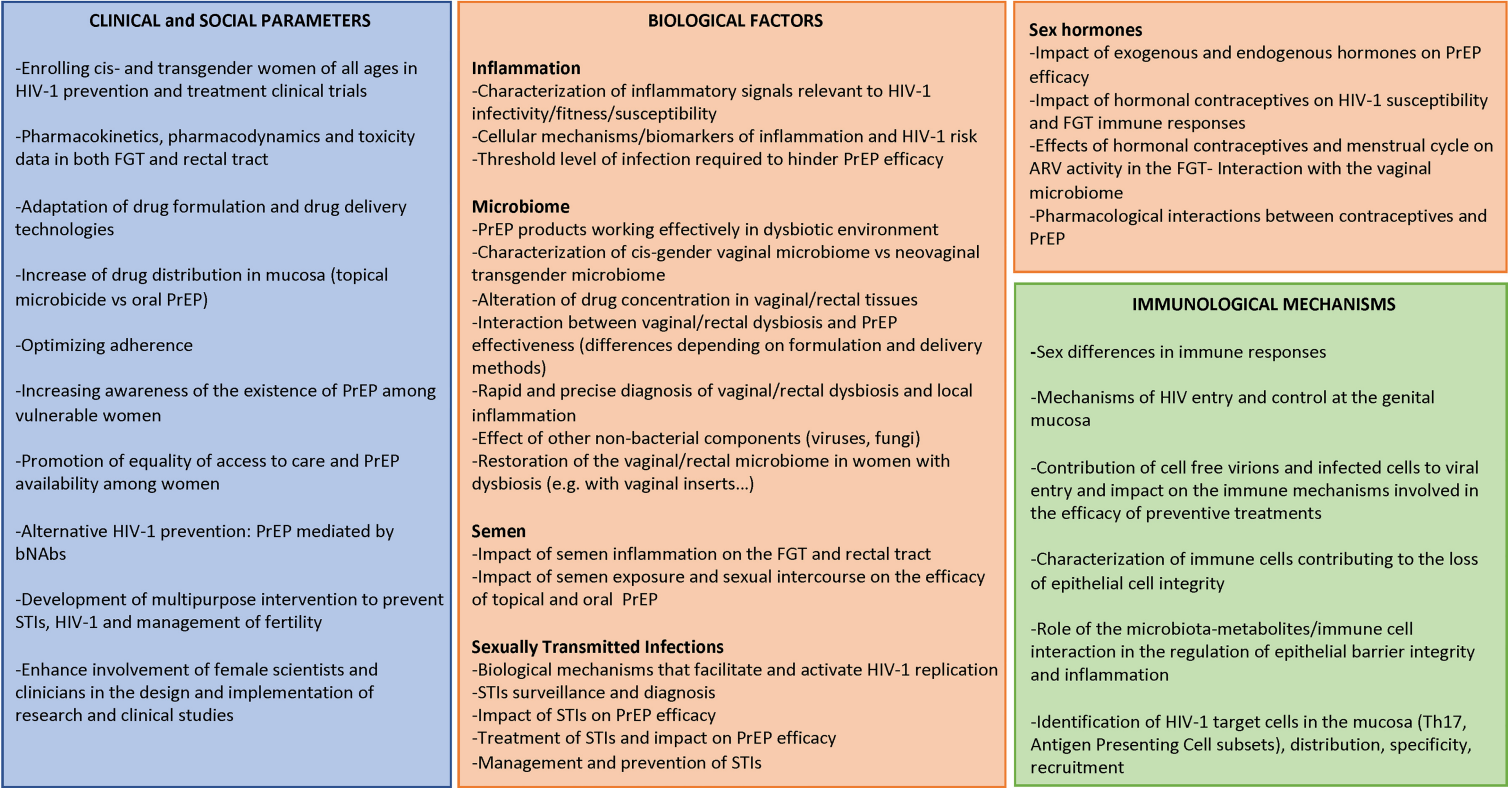
Identification of the gaps to optimize HIV-1 prevention in women. FGT, female genital tract; bNAbs, broad neutralizing antibodies; STI, sexually transmitted infections; ARV, antiretrovirals.

Given that women bear a disproportionate higher risk for HIV infection and that their biology differs from those of men, here we discuss the biological factors affecting the effectiveness of pre-exposure prophylaxis (PrEP) in women and the importance of including women in HIV prevention clinical trials. Furthermore, we discuss how the outcomes of these studies would greatly benefit from a better understanding of the immunobiological mechanisms of HIV infection and pathogenesis. To conclude, we emphasize the underrepresentation of female scientists in STEM disciplines and the important role they could play, together with advocates and regulatory bodies, in the design and implementation of HIV clinical research to advance the field more equitably (**Figure 1**). While here we mostly focused on PrEP studies conducted in sub-Saharan Africa, this article aims to give voice to women from every region of the world and to promote global women’s health.

This review has been drafted by female scientists belonging to FEMIN (a network of European female scientists and clinicians with a common interest in mucosal immunology and STIs including HIV-1) and CAPRISA (Centre for the AIDS Programme of Research in South Africa, a consortium of five institutions in South Africa and the US). Our common interest is accelerating and expanding evidence-based women-initiated HIV prevention and treatment technologies and to amplify the voices of advocates to advance the research agenda towards greater inclusivity in the knowledge generation process from conception, to the design and conduct of clinical studies and rapid implementation of efficacious products to address the needs of all people at HIV risk or living with HIV.

## Currently available HIV prevention strategies in women

The development of technologies that empower women to protect themselves from HIV remains an essential tool in the fight against the HIV epidemic, particularly in high prevalence regions such as in sub-Saharan Africa. Since July 2010, when antiretrovirals (ARVs) were first shown by the CAPRISA 004 trial (17) to prevent sexual transmission of HIV, the HIV prevention landscape has been transformed, principally through oral and topical tenofovir (TFV)-containing PrEP (18–23) including the more recent expansion of provider initiated PrEP options or through early antiretroviral therapy (ART) initiation in HIV-positive individuals (Treatment as Prevention [TasP]) (24).

### PrEP in *cis*-gender women

**Figure 2:**
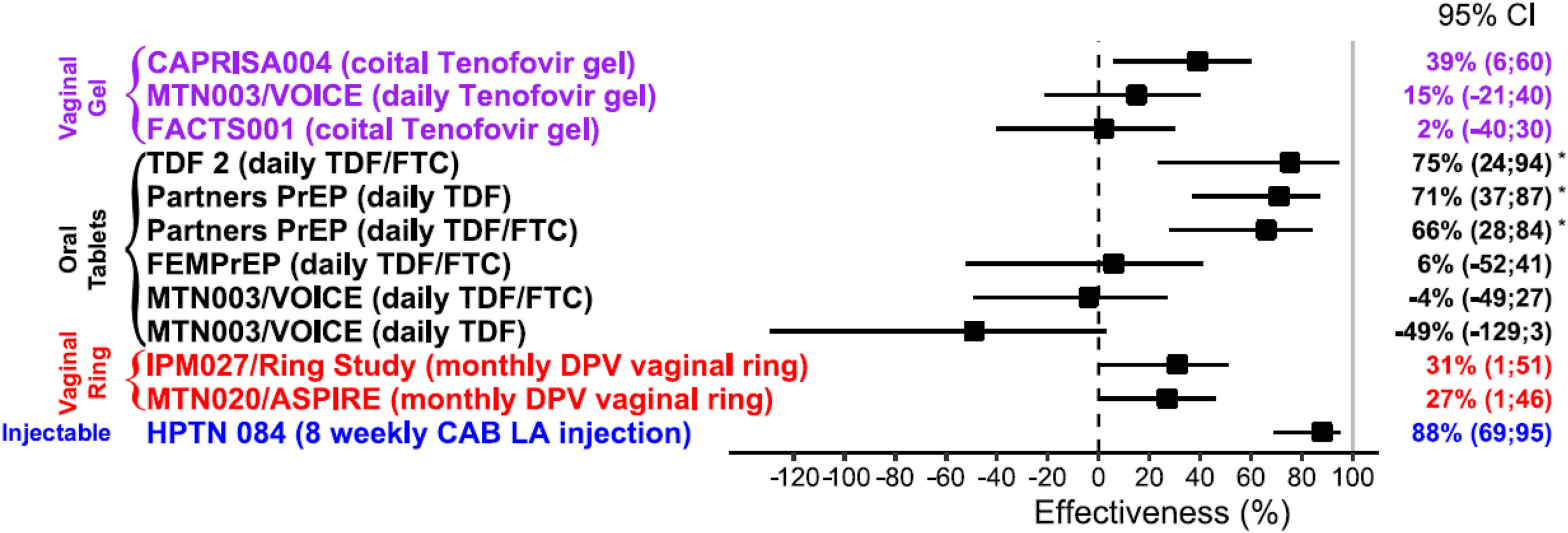
Effectiveness of PrEP in *cis*-gender women (17, 20–23, 25–28). *Subgroup analysis of effectiveness by gender, (TDF,Tenofovir disoproxil fumarate; FTC, Emtricitabine; DPV, Dapivirine; CAB LA, Cabotegravir long acting).

Efficacy data from completed trials conducted in *cis*-gender women are summarized in **Figure 2**.

#### Topical and oral PrEP

The results of clinical trials of topical and daily oral PrEP in African women have been inconsistent, most likely due to varying adherence and biology (20–23).

While the CAPRISA 004 trial demonstrated partial protection with TFV gel used before and after sex in which HIV infection was reduced by approximately 39% overall, and by 54% in women with high adherence to the protocol (17), the follow on trial FACT001 showed no difference in risk of infection between active and placebo groups (25). In a subgroup analysis consisting of 214 FACTS participants in the TFV-treated group, having high tenofovir drug levels in genital fluids was significantly associated with a 48% lower risk of HIV acquisition. In the VOICE trial none of the evaluated drug regimens reduced the rate of HIV acquisition in an intention-to-treat analysis however, adherence was suboptimal: only 25%, 29%, and 30% of women allocated to the daily TFV gel, daily oral tenofovir disoproxil fumarate and emtricitabine (TDF/FTC) and daily oral TDF groups respectively had detectable drug levels (23). While TDF/FTC provides high level of protection from rectal exposure, even with intermittent usage (29), vaginal protection appears to require dosing with greater fidelity to achieve and maintain high drug levels needed for protection. PK studies have documented significant heterogeneity in active drug concentrations between mucosal tissues that may partially explain the disparities in effectiveness between men and women. After a single oral dose of TFV, active drug concentration in the FGT was 100-fold lower compared to the gastrointestinal tissues, and it takes 7–14 days of dosing to reach protective FGT concentrations (30). Although we do not know the effective tissue concentration required to prevent HIV infection, less than 1000 ng/ml of TFV in CVF was associated with no significant protection in CAPRISA 004 (31), highlighting that the threshold for drug efficacy is higher in women (17).

The red algae-derived lectin Q-Griffithsin, when formulated as a vaginal gel, protected Rhesus macaques from a high dose vaginal challenge (32) and was safe in a phase 1, randomized, placebo-controlled first-in-human trial (33).

An in-depth knowledge of the various elements of microbicide acceptability in various cultural contexts is crucial since acceptability is likely to be a major factor in the actual use of PrEP by women, with consequences on adherence and anti-viral efficacy. This entails investigating acceptability not only in terms of product-related issues but also of women’s capacity to negotiate the use of PrEP with their partners. Results from a Phase II study of 1% TFV topical gel as part of the HIV Prevention Trials Network (HPTN) 059 suggested that microbicides were more acceptable in coitally-associated users than in daily users (34). However, on demand (event-driven) and daily PrEP in women have raised significant concerns about feasibility and acceptability affecting adherence and whether it can achieve the higher drug level threshold required in women compared to men (35).

Daily oral TDF/FTC has been demonstrated to be consistently effective in MSM and *trans*-gender women globally (18, 19) and is highly efficacious for HIV prevention among women if taken with high, but not perfect, adherence. Similarly to what observed for the VOICE trial, in the FEM-PREP trial only 24% of the women allocated to the daily oral TDF/FTC group had detectable drug levels, confirming that lack of protective effect can partially be explained by suboptimal adherence (22). In a sub-group analysis of the Partners PrEP Study conducted in women at higher risk for HIV-1 acquisition, efficacy point estimates ranging from 64% to 84%. Monthly clinic attendance for PrEP refills was > 94% and TFV detection in plasma was high (> 70%) (36). Interestingly, the Bangkok Tenofovir Study evaluating oral daily use of TDF in male and female drug users, reported 79% efficacy in women and was associated with the highest rates of adherence (66% by females) (37).

The DISCOVERY study, evaluating the combination of tenofovir alafenamide and emtricitabine (TAF/FTC), did not enroll *cis*-gender women, therefore the drug is not approved for use in this population. Considering the unfavorable results of initial clinical studies and the availability of new drugs, *cis*-gender women should definitively be included in new clinical trial of PrEP efficacy. Gilead currently has an ongoing trial evaluating the efficacy of oral TAF/FTC in adolescent girls and young women as part of their “Women’s HIV Prevention Study” (38).

Adherence and persistence are crucial and engaging women in PrEP may be particularly challenging, likely due to the difficulty of taking daily medication. In the HIV Prevention Trials Network (HPTN) 082 trial, PrEP initiation was high and half of study participants persisted with PrEP through month 12, resulting in 1% HIV incidence in this cohort  (39). Thus, while strategies to support PrEP use and less adherence-dependent formulations are needed, daily oral PrEP should remain an option for women who can use it. Approval of daily oral PrEP in the form of TDF/FTC (marketed as Truvada^®^) by regulatory authorities in South Africa, the European Union (EU), Canada, Kenya and other countries, following on from the United States (U.S.) Food and Drug Administration (FDA) approval (40, 41) and the 2015 WHO guidelines that recommend the use of TFV-containing PrEP in both men and women (42), have given impetus to the search for improved formulations to overcome adherence challenges related to daily oral PrEP. However, it also seems that women are more likely than men to require near-perfect adherence to oral TFV-containing HIV prevention therapy and may need to take 5 or 6 out of 7 doses in a week, in order to be protected against HIV (43). On-demand PrEP before and after sex is effective among MSM (44) however, findings of small blinded trials suggest low adherence to sex-event-driven dosing in African female sex workers and serodiscordant couples (45, 46). The results from the HPTN 067/ADAPT Cape Town trial investigating the feasibility of non-daily PrEP in adult women, supported recommendations for daily use of PrEP with oral TDF/FTC (47). On-demand oral PrEP is currently not recommended in women due to the lack of efficacy data and concerns about the higher adherence needed for women.

#### Long-acting topical PrEP

To address the daily adherence challenge, attention has turned to development of longer acting slow release and/or provider initiated PrEP options including monthly tablets or vaginal rings, 2-6 monthly injections and annual implants. Thus far proof of concept trials have been completed for the monthly vaginal ring and a two monthly injectable. In the ASPIRE dapivirine (DVR) vaginal ring trial, where women were provided with a monthly vaginal ring, the incidence of HIV-1 infection in the dapivirine group was lower by 27% (95% confidence interval (CI), 1 to 46; p=0.046) than that in the placebo group (26). In a *post-hoc* analysis, higher rates of HIV protection were observed among women over the age of 21 years (56%; 95% CI, 31 to 71; p<0.001) but not among those 21 years of age or younger (-27%; 95% CI, -133 to 31; p=0.45), a difference that was correlated with reduced adherence (26). In the RING trial the incidence of HIV-1 infection was 31% lower in the dapivirine group than in the placebo group (hazard ratio, 0.69; 95% CI, 0.49 to 0.99; p=0.04), however there was no significant difference in efficacy of the dapivirine ring among women older than 21 years of age compared to those 21 years of age or younger for treatment-by-age interaction (27). A secondary analysis from these studies indicated the potential for a substantial reduction in the risk of HIV-1 acquisition – greater than 50%, and potentially as high as 75% to 91% – in women who consistently use the ring (48). The WHO formulated a conditional recommendation supporting the use of dapivirine ring in women and found that “potential benefits outweigh the harms based on a systematic review and meta-analysis of the scientific evidence including data on cost–effectiveness, acceptability, demonstrated feasibility, and the potential to increase equity as an additional prevention choice, noting some variability in effectiveness in younger age groups and limited data regarding use among pregnant and breastfeeding women” (49). DVR has been formulated also in the form of an extended-release monthly film (https://matrix4prevention.org/products/extended-release-dapivirine-vaginal-film).

#### Multi-month injectables

A long-acting injectable form of cabotegravir (CAB LA), a two monthly injectable has recently been approved by the United States Food and Drug Administration (FDA) and EMA for its PrEP indication. Tested in *cis*-gender women for prevention efficacy in the HPTN 084 trial, HIV infection risk was 88% lower in the cabotegravir group compared to TDF-FTC (28). These findings were consistent with results from the HPTN 083 trial, which was conducted in *cis*-gender men and *trans*-gender women who have sex with men (50). However, both these well tolerated phase 3 trials were stopped early due to high levels of efficacy as evidenced by fewer incident HIV infections in participants receiving long-acting cabotegravir every 8 weeks compared with daily oral TDF/FTC for PrEP. Despite these successes and the potential for CAB LA to overcome adherence challenges, concerns about cost and access to low- and middle-income countries (LMICs) remains a barrier to use for both treatment and prevention indications (51). ViiV is currently in discussions with the Medicines Patent Pool and several generic drug manufacturers for low-cost production of their two monthly injectable for LMICs. The recent IAS conference held in Montreal saw substantial discussions and advocacy efforts to address the access challenges that was primarily evaluated using public sector funding.

Trials of 6 monthly injectables, monthly pills and annual implants are ongoing (https://www.avac.org/).

### PrEP in *trans*-gender women

Fewer studies have investigated PrEP efficacy in *trans*-gender women, despite the high rates of HIV infection in this population. In a sub-group analysis of the iPrEX study, among 296 participants identified as *trans*-gender there were 11 HIV infections in the PrEP group and ten in the placebo group. However, no drug levels were detected in the *trans*-gender women at the seroconversion visit (52).

More recently, the combination of TAF/FTC has been approved as PrEP for both MSM and *trans*-gender women, following the results of the DISCOVER study which showed that TAF/FTC was non-inferior to TDF/FTC for HIV prevention (IRR 0·47 [95% CI 0·19-1·15] at the time of the primary efficacy analysis) (53).

The possible interaction between gender-affirming hormone therapy (GAHT) and PrEP drugs, leading to a lower PrEP efficacy in *trans*-gender women is a potential concern that can only be addressed by trials that include *trans*-gender women on GAHT. Data from PrEP trials completed to date on antiretroviral interactions with GAHT have been reassuring. Results from a sub-study in the Phase III DISCOVER trial demonstrated that drug levels in peripheral blood mononuclear cells (PBMC) were comparable between *trans*-gender women on GAHT and MSM receiving TAF and between *trans*-gender women on GAHT and MSM receiving TDF (54). In a recent study with *trans*-gender women and *trans*-gender men after four weeks of daily TDF/FTC use, PrEP did not affect estradiol or testosterone levels. Conversely, GAHT did not affect the concentration of PrEP drug levels in the blood, and all *trans*-women and trans men in the study had drug levels expected to provide high protection from HIV (55). In addition to providing reassurance to individuals taking gender-affirming hormones with daily PrEP, the study opens the possibility of PrEP on demand for *trans*-individuals taking hormones who may be exposed to HIV by anal sex.

Additionally, data from the HPTN 083 trial presented at the AIDS 2022 conference suggested that there is no impact of GAHT on CAB LA concentrations (56)

### PrEP in pregnant women

High rates of incident HIV infection have been reported in pregnant women in the last trimester and post-partum (57) with implications for vertical transmission. Pregnant women are among those the most in need of safe HIV prevention treatments however, pregnancy has been an exclusion criterion for large trials of PrEP (17, 21, 58), mostly because of fetal safety concerns (59). For the same reason, women of reproductive age are often excluded from clinical trials of antiretroviral treatments or prevention strategies. This exclusion has generated critical knowledge gaps in optimal dosing during pregnancy, fetal safety and maternal outcomes (60). Pharmacokinetics (PK) data specific to pregnancy would inform on the dose required to protect the women and their offspring, while avoiding their exposure to drug related toxicities or undertreatment (60). In the Partners PrEP study, women who received TDF or TDF/FTC at the time of conception did not differ from those who received a placebo in terms of pregnancy incidence, birth outcomes, or infant weight. A recently completed nonrandomized, phase-IV prospective study of darunavir and cobicistat PK in pregnant women with HIV and their children in the U.S. reported significantly lower exposure during pregnancy, which may increase the risk of virologic failure and perinatal transmission (61). Daily oral PrEP (TDF/FTC) is now widely used by adolescents and pregnant and lactating women. A randomized trial of safety and PK of the dapivirine vaginal ring versus FTC/TDF when used during pregnancy is planned (https://mtnstopshiv.org/research/studies/mtn-042). These studies are of utmost importance, considering that the risk of HIV acquisition among women increases during pregnancy and the postpartum period (57).

### PrEP and hormonal contraception

Effective contraception and prevention of sexually transmitted infections (STIs), including HIV-1, are global priorities for women that are ideally addressed through comprehensive sexual reproductive health (SRH) service provision and the development of multi-purpose technologies that meet HIV, STI and fertility control needs of users. In most clinical trials of PrEP *cis*-women participants are required to be on a reliable form of contraception. Notwithstanding, pregnancies do occur and have enabled some safety data of PrEP use during pregnancy to be generated. In FEM-PrEP, the group of women receiving TDF/FTC experienced more incident pregnancies than the placebo group, although this difference was not statistically significant when baseline characteristics were taken into account (62). An analysis from the Partners PrEP study reported that pregnancy incidence among women who used oral contraceptives was comparable to that among those who did not, and this lack of contraceptive effectiveness was similar for those assigned PrEP and placebo. Injectable or implantable hormonal contraception plus PrEP provides effective prevention for pregnancy and HIV-1 (63). However, women on combined oral contraceptives were also less likely to adhere compared to injectable users. While pharmacokinetic interactions between TDF/FTC and hormonal contraceptives are not anticipated, pharmacodynamic and/or behavioral interactions could affect the results of this kind of studies.

African adolescent girls and young women (AGYW) starting PrEP have high rates of asymptomatic, curable bacterial STIs (64, 65) making syndromic STI case management ineffective and necessitating the use of rapid, sensitive and affordable STI testing that is being enabled through point of care diagnostic technologies. The distribution of STIs, as well as HIV incidence and prevalence, is shifted toward younger age groups for females in comparison to males (66). Thus, the urgent need to prioritize and identify groups of AGYW who are most vulnerable to HIV and other STIs.

### PrEP in menopausal women

Clinical trials evaluating PrEP in women have so far primarily targeted younger females however, menopausal women represent another vulnerable population. Menopause and increasing age do not result in a reduction in sexual activity, but can have an impact on how much people perceive themselves to be at risk of HIV infection. Both in the U.S. and in highly endemic regions like South Africa, older adults frequently engage in high-risk behaviors like unprotected sexual activity (67–69). This may be because they are less concerned about pregnancy protection after menopause and underestimate the risk of STIs. Age-related comorbidities, particularly cardiovascular disease, neurocognitive dysfunction, and bone mineral disease are all potentially heightened by HIV or its treatment. Of note, women with HIV experience more vasomotor symptoms than women without HIV around the time of menopause (70, 71), which is associated with reduced adherence to ART and thus, suboptimal clinic attendance (72). In addition, several biological risk factors such as vaginal pH, epithelial function, target cell frequency and innate immunity are altered during menopause (73–75). All those factors may have an impact on PrEP efficacy. Using *ex vivo* cervical tissues from premenopausal and postmenopausal women, active metabolite concentrations of tenofovir and emtricitabine were lower in postmenopausal explants, indicating that postmenopausal women need higher doses of tenofovir-based PrEP to achieve protective efficacy (76). These data need to be validated *in vivo*.

## Challenges to effective prevention strategies in women

### Access to health services

To realize the full impact, PrEP requires access and adherence. For women in Africa, access to medical care is not always optimal. Women are required to be compliant with daily oral PrEP and regular visits to the clinic may become challenging. Women also face stigma for possession of ART, particularly because the same drugs are used for both HIV treatment and prevention. This associated stigma may lead to discontinuation and non-adherence, impacting efficacy. A necessary pre-requisite for PrEP use is a good perception of risk. Perception of risk particularly in young women transitioning to adulthood is low.

### More provider-initiated PrEP technologies

There is a real need to adapt drug formulation and to innovate drug delivery technologies to encourage uptake and adherence. The PrEP landscape has expanded substantially in the past decade with a focus on slow release long acting agents. Recent promising advances in the field include lenacapavir, a long-acting HIV capsid inhibitor that demonstrated viral suppression in both ART naïve individuals and individuals who had multi-drug resistant virus. This six-monthly injectable is currently being assessed in HIV prevention clinical trials (77–79). The oral islatravir nucleoside reverse transcriptase translocation inhibitor, administered monthly and an islatravir subdermal implant administered annually is currently being evaluated as PrEP options (80). Of note is that the FDA halted all islatravir studies in December 2021 due to unexpected findings of declines in total lymphocyte and CD4+ T-cell count in some study participants, although the mechanism and duration of effect of lymphopenia is not clear yet (81). The lymphocytopenia was transient and normalized once the islatravir was discontinued, however the risk of depleted lymphocytes predisposes to increased risk for infections. Under early stage clinical evaluation is also an annual TAF implant and an elvitegravir (EVG)/TAF vaginal insert (35, 82, 83). Preclinical proof-of-concept for preventing vaginal transmission of simian human immunodeficiency virus (SHIV) in non-human primates has been demonstrated for this product (35).

### Enhancing choice PrEP to meet diverse needs/increasing drug levels at the point of exposure to HIV

The use of topical microbicides, in the form of vaginal or rectal inserts, gels or rings, may meet the needs of individuals for whom the use of oral or injectable daily PrEP is not acceptable. The development or refinement of such products is a priority in the field (84) and different formulations, including vaginal films and tablets, are in later-phase preclinical development (https://www.avac.org/infographic/future-arv-based-prevention). Topical inserts may be designed to quickly disintegrate or dissolve within the vagina or rectum to release therapeutic drugs quickly for event-driven use, or they may provide an extended/controlled drug release through bioadhesive properties that may prolong retention and pharmacokinetics, depending on the type of formulation technology used.

Topical microbicides have the advantage, over oral PrEP, to release the drug in the anogenital tissues (both the female genital tract (FGT) and rectal tissues)and fluids, potentially providing protection at the site of virus transmission. Indeed, TFV levels in the cervico-vaginal fluids (CVF) and in genital tissues are more than 100-fold higher after vaginal gel insertion than after oral tablet ingestion (30, 31, 85–87). Protection may require both systemic and mucosal concentrations in a combination that differs between oral and topical dosing, and the use of preclinical models could be very informative in that sense. The R-PrEP trial, comparing the PK of raltegravir and lamivudine in genital tissue against *ex vivo* tissue infection with HIV-1, demonstrated that after 7 days of raltegravir dosed with or without lamivudine twice a day, protective levels were reached at different times in the FGT and the rectum, and that different PK and PD profiles were observed after dosing cessation (88). In spite of the high efficacy of CAB LA, pharmacodynamics (PD) data indicated low drug levels in the genital tract, suggesting that reaching sufficient drug concentration in the regional lymphoid tissue might be more important than topical genital levels (89).

Moreover, drug distribution in different mucosal cells needs to be considered. *In vitro* studies using human tissues from the upper and lower FGT suggested that concentrations of active TFV were 100 times higher in epithelial cells than in CD4+ T cells (90), and imaging methods in animal vaginal tissues were used to confirm the preferential TFV concentration in the epithelium (91). Additional studies showed that after first TFV treatment, endometrial, endocervical and ectocervical polarized epithelial cells secreted TFV that partially protected CD4+ T cells *in vitro* for at least 3 days (92). Importantly, luminal epithelial secretions were not protective, but TFV release was specific towards the basolateral epithelial compartment. This suggests that when TFV concentrations are below ideal levels, factors that increase target cell presence on the mucosal surface, such as inflammation or damage, would overcome the TFV protective effects. This concept is supported by CAPRISA 004 clinical data describing ideal conditions for increased intracellular metabolite competition and reduced drug efficacy in the context of inflammation and immune cell activation (93).

Rectal HIV transmission to women is an under-studied area in HIV research, although a largely overlooked fraction of men to female transmission is due to anal intercourse (94–97). It is recognized that women engage in receptive anal intercourse and often due to violence (98). While it is possible for products applied vaginally to reach the rectum, as shown in non-human primate (NHP) models (99), it is unknown if these levels offer protection against anal HIV acquisition. In addition, both PK and PD of drugs differs by gender and needs to be defined in both the FGT and the rectal tract. The interpretation of conflicting trial results may be affected by differential drug levels in the vagina and rectum as well as sexual behaviors across research participants. The risk of contracting HIV during one receptive anal act without PrEP or condoms use is about 10 times higher than the risk during one receptive vaginal act without PrEP or condoms (97, 100, 101). Q-Griffithsin was also assessed as a rectal microbicide, as part of the PREVENT program and the *in vivo* effect on mucosal cell populations was evaluated in Rhesus macaques. The study indicated that despite multiple application of Griffithsin, the frequencies of rectal E-cadherin+ cells remain stable, whereas a minor increase in the frequencies of rectal mucosal CD4+ cells was observed (102). A phase 1 clinical trial of Q-Griffithsin against rectal HIV transmission is ongoing in adult men and women and will inform on the safety and pharmacokinetics of this microbicide (https://clinicaltrials.gov/ct2/show/NCT04032717). The efficacy of TAF/EVG inserts as topical on-demand rectal PrEP was demonstrated in non-human primates (103). The Microbicide Trail Network (MTN) 039 is an ongoing phase 1 open label safety and PK study of single dose rectal administration of a TAF/EVG insert (https://www.mtnstopshiv.org/research/studies/mtn-039).

## Biological factors influencing HIV transmission and the efficacy of prevention strategies in women: what do we know and where are the gaps?

While adherence is a key factor to guarantee efficacy of PrEP trials, it is becoming increasingly evident that several biological factors can modify microbicide efficacy in women. Those factors should be considered when planning both PrEP interventions and when analyzing safety and efficacy of treatments.

### Genital inflammation

It is well known that genital inflammation plays a significant role in the susceptibility to HIV infection (104). The risk of contracting HIV has been linked to epithelial disruption, elevated levels of pro-inflammatory cytokines and chemokines in genital secretions and the activation or recruitment of HIV target cells (105–111). Genetic factors, vaginal microbial diversity, intravaginal practices, sexually transmitted infections, semen exposure, endocrine sex hormones, and hormonal contraceptives are some of the factors linked to genital inflammation (111–114). However, the type of inflammatory signals that are more relevant to HIV infectivity/fitness and/or susceptibility are not known and need to be investigated.

Genital inflammation has been clearly implicated in diminished protection by topical TFV. McKinnon et al. showed TFV to be 57% protective against HIV in women without vaginal inflammation but only 3% protective if genital inflammation was present. Among women who used the gel consistently, TFV protection was 75% in women without inflammation compared to -10% in those with inflammation (115). An additional significant factor that may modify protection following topical application is the potential ability of TFV to delay wound healing, as shown *in vitro* (116). According to the results of the Progect Gel phase I clinical trial, rectal administration of 1% TFV gel caused considerable changes in the proteome of epidermal cells, including extracellular matrix, tissue remodeling, epidermal growth, tight junctions, and cellular stress pathways (117). Additionally, the development of vaginal ulcers forced the termination of a recent clinical trial investigating the safety of a tenofovir disoproxil fumarate ring in healthy women (118).

While inflammation has been independently associated with HIV risk and CD4+CCR5+ T cell target concentrations, a direct cellular mechanism for the relationship between HIV and inflammation has not been established and a cellular biomarker of HIV risk remains unconfirmed. The ability of endometrial stromal fibroblasts to facilitate HIV acquisition by *trans*-infection (119) is exacerbated in conditions of genital inflammation however, the threshold level of infection required to undermine the efficacy of PrEP, especially in women with inflammation, is unknown.

### Genital microbiome

The microbiome that is considered a key regulator of genital inflammation and vaginal dysbiosis, has been linked to HIV risk (108, 110, 120). This poses a serious public health concern given that bacterial vaginosis is widespread in areas with a high prevalence of HIV. Several studies have shown that a vaginal environment dominated by some Lactobacillus species promotes vaginal acidity, anti-microbial defense, and an anti-inflammatory tissue environment, and has been consistently associated with healthy pregnancy outcomes, lack of abnormal vaginal symptoms and reduced risk for acquiring HIV and other STIs (121–123). Vaginal dysbiosis results in an increased number of activated CCR5+ CD4+ T cells in the vaginal mucosa, and higher production of pro-inflammatory cytokines in the cervical secretions (110, 124). Women having the highest concentrations of *Lactobacillus crispatus* had a decreased HIV acquisition risk relative to those dominated by *Gardnerella vaginalis* and other anaerobes (125). However, women having a vaginal microbiome dominated by *Lactobacillus iners* had similar incidence of HIV compared to women having dysbiotic microbiota. The FRESH Cohort study, enrolling 200 South African women aged 18-23 years old, showed that HIV-1 risk acquisition increased markedly with increased dysbiosis, and remarkably, none of the women whose microbiota was dominated by *Lactobacillus crispatus* acquired HIV-1 throughout the one year of the study (125).

PrEP products that will work effectively in an environment of vaginal dysbiosis are needed. Furthermore, an understanding of the similarities and differences in the vaginal microbiome of *cis*-gender and the neovaginal microbiome of *trans*-women would inform the development of appropriate targeted strategies (126). Indeed, it has been shown that the genital microbiome influences the effectiveness of TFV gel and dapivirine, with higher protection in women whose vaginal microbiomes is dominated by Lactobacilli than in women with vaginal dysbiosis (125, 127). Although the methods used were sometimes insufficient to distinguish Lactobacillus communities dominated by beneficial species such as *Lactobacillus crispatus* versus less beneficial species such as *Lactobacillus iners*, they raised concerns that TFV-based microbicides could be undermined by microorganisms like *G. vaginalis* or *Prevotella* species, which could effectively reduce the level of drug available locally. Also, in women using TFV vaginal gel or vaginal film, lower levels of TFV and the active metabolite, tenofovir-diphosphate (TFV-DP), were found in the genital tissues of those with higher concentrations of *G. vaginalis*, *Atopobium vaginae*, or a diagnosis of bacterial vaginosis (128). It is possible that declines in drug levels might be countered by increasing the amount of drug supplied or by using continuous release drug delivery platforms, like a vaginal ring. In support of the latter hypothesis, a study demonstrated no changes in the level of tenofovir-diphosphate in women wearing TFV vaginal rings stratified by Lactobacillus dominating versus dysbiotic vaginal community states (129). Results from *in vitro* studies aiming at understanding the mechanisms through which the vaginal microbiota might alter the drug concentrations in genital tissues, lead to the hypothesis that *G. vaginalis*, which is often present in greater quantities among women with non-Lactobacillus dominant microbiota, metabolizes TFV to adenine or secretes adenine which is consumed by *A. vaginae*, creating bacterial synergy between these species in the vaginal ecosystem (125, 127). TFV metabolism can occur quite quickly. In the presence of high concentrations of *G. vaginalis* TFV-DP levels were reduced within two hours in cervical tissue and in one week in CVF and plasma (125, 128). It has been also reported that dapivirine binds irreversibly to bacteria *in vitro* (127), thus impacting the availability of the drug to diffuse into the genital tract tissues. Retrospective study of the Partners PrEP study, however, did not uncover any appreciable variations in effectiveness (130). There are still unanswered questions regarding the biological mechanisms of the potential interaction between vaginal dysbiosis and PrEP effectiveness in preventing HIV acquisition, as well as whether this relationship differs depending on the formulations (i.e. TFV-, dapivirine-, etc) and delivery methods of PrEP (such as oral, topical).

A regular collection of vaginal swabs and mucosal biopsies may allow to better examine the relationship between PrEP effectiveness and changes of the microbial community. Since there are increasing indications that vaginal bacteria can modify topical TFV efficacy (125, 131), additional studies determining if a particular bacterial species or the diversity of bacterial communities as a whole are linked to decreased efficacy of new PrEP candidates are warranted. A rapid and precise diagnosis of vaginal dysbiosis and local inflammation, although challenging and not everywhere possible, will be critical in improving the efficacy of HIV prevention efforts in women and to improve public health policy (132). Furthermore, the effect of other non-bacterial components of the microbiome such as viruses and fungi on the efficacy of PrEP need to be investigated. Several efforts are underway to evaluate vaginal inserts to restore the vaginal microbiome in women with dysbiosis (133).

### Semen

Semen in addition to being a carrier of HIV is also an important modulator of genital inflammation and immune activation at both the FGT (134–136) and the rectal mucosa (137, 138). The production of pro-inflammatory and chemotactic cytokines is increased in the female genital mucosa in the presence of semen (139–145). Several of these cytokines are linked to leukocyte recruitment (141, 144–146) and decreased mucosal barrier integrity (147, 148), both of which play a significant role in HIV’s ability to enter and access target cells at the mucosal sites. The impact of the level of inflammation in the semen on the female genital tract and rectal mucosa needs further investigations. In addition, studies indicated that semen exposure and sexual intercourse may promote a shift in the microbial environments of the FGT, inducing bacterial vaginosis, that may facilitate HIV infection in women (134, 149–151). On the other hand, semen has been shown to be able to prevent viral entrance into the genital mucosa, because of the presence of several inhibitory factors like cationic polypeptides and reactive oxygen species with anti-HIV activity (152, 153). Importantly, semen exposure and sexual intercourse may also affect efficacy of topical PrEP (154–156) by redistributing cervicovaginal fluid and topically applied microbicides (157, 158). After coitus, tenofovir gel concentrations were noticeably lower in CVL, cervical tissues, and vaginal tissues (155), and in *in vitro* studies a significant reduction in the antiviral activity of PRO2000 gel was observed post-coitus (156).

### Sexually transmitted infections

STIs and HIV share a complex, synergistic bidirectional relationship (159, 160) and are associated with an increased risk of HIV acquisition and transmission at both the FGT and rectal tract, mainly linked to a high inflammatory environment (161–164). Evidence suggests that for HIV positive individuals, persistent high risk behaviors increase susceptibility to STIs, and advancing HIV infection may increase the frequency of STIs treatment failures (161, 162). Conversely, asymptomatic or symptomatic STIs strongly predict susceptibility to HIV, augment HIV shedding at genital mucosal sites and increase infectiousness from HIV positive individuals. Potential biologic mechanisms that facilitate and activate HIV replication include alterations in the genital tract microbiome, localized inflammation, recruitment of CD4+ T-cells, monocytes, Langerhans’ cells, and increased levels of interleukin-10 (165, 166). Recent studies have delineated the role and biologic mechanisms of STIs with disturbances in the vaginal microbiome, inducing mucosal inflammation, yielding unique cytokine profiles that evoke an influx of HIV receptor cells in genital mucosal epithelium that has been found to be associated with almost a three-fold increase in HIV viral shedding potentially enhancing sexual transmission of HIV (166). Thus, treatment of STIs remains a public health priority to potentially reduce STIs related HIV acquisition and transmission. In the population-based survey undertaken in rural and peri-urban KwaZulu-Natal, the province with the highest HIV prevalence in South Africa, just over half of participants in the study area had HSV-2 infection and just under one-quarter had at least one curable STIs of syphilis, *N. gonorrhoeae, C. trachomatis, T. vaginalis* and/or *M. genitalium* (167). Estimates of curable STIs were higher in the younger age groups, particularly in younger females, which is the same group among whom a peak in HIV incidence occurs (168). This study also found an association of HIV positive status and curable STIs (167). Whilst ART benefits HIV positive individuals by achieving HIV viral suppression to prevent onward transmission, ART itself has no impact on STIs (169) and STIs often remain untreated contributing to transient or intermittent HIV viral shedding in genital secretions (162, 170), thus the sustained potential elevated risk of onward HIV transmission. These findings highlight the importance of enhanced STIs surveillance and investment in targeted STI control programs for younger populations. STIs also impact efficacy of PrEP by damaging the vaginal epithelium or through other immunological mechanisms that need to be further investigated. In CAPRISA 004 women with high cytokine levels, as is typical of STIs, experienced a reduction in the efficacy of TFV gel (17). On the other hand, TFV gel in CAPRISA 004 was 51% protective against HSV-2 acquisition (17) and Andrei et al. demonstrated that the concentration achieved intravaginally with a 1% TFV topical gel has direct anti-herpetic activity (171). The Partners PrEP study additionally demonstrated that daily oral TDF-based PrEP significantly reduced the risk for HSV-2 acquisition among heterosexual men and women (172). It was also shown that PrEP differentially altered the activation of circulating versus mucosal immune cells in HSV-2 seropositive women (173). Griffithsin, which is currently under evaluation at the University of Pittsburgh as rectal microbicide against HIV rectal transmission, is a highly potent viral entry inhibitor with broad activity against HSV-2, HCV, and HPV (32, 174). The CONRAD’s topical insert combining TAF with elvitegravir (EVG), an integrase inhibitor, provides a wide pre- and post-coital window of prophylaxis against both HIV and HSV (35). How STIs treatments might impact PrEP efficacy is an open question.

### Sex hormones

Hormonal changes during the menstrual cycle induce several modifications of the immune system and of the microbiota composition within the FGT, affecting the susceptibility to HIV and other STIs. The immune protection is dampened during the secretory phase of the cycle to optimize conditions for fertilization and pregnancy, and the progesterone-high phase of the menstrual cycle is indeed a risk factor for HIV acquisition (175). Furthermore, during menses, the local vaginal inflammation increases and the vaginal microbiota is disturbed (176).

The impact of women’s exogenous and endogenous hormones (including the hormone replacement therapy (HRT) in *trans*-gender women) on the efficacy of PrEP and their risk of contracting HIV deserves further investigations. Through a number of biological mechanisms, including changes in gene expression, progesterone-induced epithelium thinning, microbiome alterations, increased HIV target cells in the FGT, hormonal contraceptives such as medroxyprogesterone acetate (DMPA), which is widely used in sub-Saharan Africa, may enhance susceptibility to HIV (177–183) and impact FGT immune responses in women. According to a *post-hoc* analysis of the Partners PrEP study, DMPA users still received protective benefit from oral TDF/FTC or TDF alone however, there was an observed 10% increase in HIV incidence in women using hormonal contraception compared to those who didn’t used DMPA (184). Although the ECHO trial did not find a substantial difference in HIV risk among women using DPMA as a contraceptive (185), this study had several limitations and more research is required to better understand the effects of hormonal contraceptives and of the menstrual cycle on antiretroviral activity in the vaginal tract as well as possible pharmacological interactions between hormonal contraceptives and PrEP (186–188).

There is emerging evidence that gender and sex hormones can play a role in the interplay between the microbiome and gastrointestinal diseases (189, 190). Since the gut microbiome is involved in the excretion and circulation process of estrogen and androgen, the concept of “microgenderome” indicating the interaction between sex hormone and the gut microbiota has been recently suggested and represents an emerging research topic (191, 192).

### Sex differences in immune responses

Sex differences have been implicated in outcome from vaccines and infections in adults and children (193, 194). In COVID-19 pandemic we have been able to clearly observe sex differences in the immune response to the virus, and how these translate into clinical outcomes (195). Differences in the acquisition of HIV and in disease pathogenesis between men and women have long been also reported in epidemiological studies (196, 197).

The X chromosome encodes the most immune-related genes of any chromosome (198) and significant sex differences in innate and adaptive immune responses have been described (199). The important genes reported to locate on the X chromosome that play a role in immunity include CD40L, CXCR3 and Foxp3. These genes are crucial for B cell maturation and antibody response regulation, tissue effector T cell recruitment and immune tolerance (200–202). Toll like receptor 7 (TLR7) is the most described gene-driven production of interferon (IFN-I) by plasmacytoid dendritic cells (pDCs) (203, 204). Seminal studies from infected macaques with simian immunodeficiency virus (SIV) point to pDCs as the critical IFN-I producers in this process (205, 206), and human studies univocally demonstrated that compared to men, pDCs from women produce more IFN-α/β in response to TLR7 ligands, resulting in higher induction of interferon-stimulated genes (ISGs) (207–210).

There is also an increased risk of *in utero* infection among females. Sex-specific innate immune selection of HIV *in utero* is associated with increased female susceptibility to infection (211). In Hepatitis C virus (HCV) infection, female infants are twice as susceptible to mother-to-child transmission (212, 213). In congenital cytomegalovirus (CMV), females have 3-fold more neurological disease (214). All these studies highlighting the need for a gender-diverse inclusion in immunological research (204). The inclusion of sex differences in the analysis of immunological and clinical data will lead to a better understanding of the immunobiology of HIV disease.

A better understanding of the immunobiological mechanisms of HIV infection, co-infections and pathogenesis, at the level of both the FGT and the rectal tract, could provide direction toward developing strategies for prevention in women.

## Identification of the immunological mechanisms to be investigated to inform prevention treatment development

In contrast to studies in MSM populations to understand pathogenesis of HIV infection and studies of vertical transmission that informed targeted and effective interventions, pathogenesis studies in women globally have been more limited and patchy.

Considerable effort by the research community has advanced our understanding of the early events of HIV genital transmission and of the initial interactions between the virus and the mucosal immunity. However, several knowledge gaps stem predominantly from challenges related to the practicality of appropriate, standardized and timely mucosal sampling in large studies and clinical trials. This limits the understanding of the effects of HIV treatment in women, and our ability to address gender differences in HIV treatment and prevention outcomes. In order to fill those gaps, we need a better understanding of the mechanisms of HIV entry at the genital mucosa and of the factors that limit this. Here, we aim to highlight, without prioritization, the immunological factors that need to be studied to address this paucity of knowledge, with the translational belief that understanding these biological mechanisms is key for product development.

An important factor to consider in early mucosal HIV transmission, and that has been mostly overlooked, is the source of transmitted virus, whether it comes from cell-free virions or infected cells. Both cell-free virions and HIV-infected cells (T lymphocytes and macrophages) have been identified in the female genital secretions and semen from HIV-infected men and have been shown *in vitro* and *in vivo* in animal models to interact with epithelial tissue and transmit infection (137, 143, 215, 216). However, HIV transmission by cell-associated HIV seems to be more efficient than by cell-free virions in male, female and anorectal mucosa (217–220). Yet, most research is done using cell-free virus inoculum and we still do not know what is the relative contribution of cell-free virions and infected cells to viral entry. Different modes of transmission may also have implications in terms of the types of immune mechanisms playing a role in protection, with an impact on the efficacy of preventive treatments.

The genital and rectal mucosal epithelia represent one of the first barriers during sexual intercourse. It is well known that during HIV infection, the gastrointestinal epithelial barrier (EB) is severely compromised, leading to translocation of microbial product into the systemic circulation, with consequent immune activation and inflammation establishment (221). Microbial translocation is considered a leading cause of inflammation in treated and untreated HIV infection. Mechanisms contributing to EB loss have not been fully elucidated, although a number of processes have been identified. Epithelial cells tight junction downregulation by HIV proteins and by inflammatory cytokines was observed using primary cells isolated from the human FGT and CD4+ T cell depletion in the gut of NHPs during acute infection was vastly documented (222–224). This included the loss of Th17 and Th22 cells, cells that are thought to function as innate regulators of mucosal integrity (225–228). In SIV infection, immunological disorders, such as Th17 depletion, happened later than changes to the structural proteins of the epithelium and microbial translocation (229). Whether other immune cells contribute to the loss of the EB during HIV infection, has been less explored. Recently, a role for intestinal CX3CR1+ mononuclear phagocytes and NKG2a/c+CD8+ T has been proposed in the protection of the gut barrier function to avoid intestinal inflammation during SIV infection (230, 231). While those cells are implicated in the EB protection also in the FGT is unknown. Bacterial metabolites such as D-lactic acid, are differentially produced from various Lactobacillus species (232). For instance, *Lactobacillus* spp. that specifically produce D-lactic acid were associated with long-term protection against *Chlamydia trachomatis* infection, consistent with reduced protection associated with *Lactobacillus iners*, which does not produce this isoform (233). The direct effect of protection was noted when FGT cells were first primed *in vitro* with D-lactic acid in contrast to adding the D-lactic acid to *Chlamydia trachomatis* first, which conferred no effect on the bacteria. These data demonstrate that the bacterial metabolites act on the cells directly rather than on the microbiota. However, further studies are needed to unravel how those mechanisms contribute to break-down of the EB and establishment of genital and generalized inflammation and potentially affect the efficacy of prevention strategies. Furthermore, the specific role of the microbiota/immune cells interaction in the regulation of those processes needs to be further explored. Therapeutic interventions aiming to promote barrier integrity and to reduce microbial translocation-induced inflammation are essential to improve the prognoses of infected individuals.

Another priority for the research community is the identification of HIV target cells in the mucosa and of the mechanism(s) contributing to their recruitment. Whether the inflammation that drives HIV acquisition risk is due to an increased frequency of CD4+ cells at the mucosa, remains an unanswered question. The presence of susceptible target cells, such as CD4+ T cells and in particular memory CD4+ T cells expressing high levels of CCR5, is a critical determinant in the establishment of HIV infection at mucosa (234, 235). Nevertheless, during the early phases of viral transmission, not all CD4+ T cell subsets are equally infected. In the SIV infection model, the Th17 were the primary targets during both vaginal and rectal transmission, together with immature DCs in the latter case (225, 236). In support of this, HIV infection in female sex workers from Kenya resulted in significant depletion of mucosal Th17 CD4+ T cells compared to uninfected samples (235). However, preferential infection of Th17 cells in humans has not been definitively demonstrated. In addition, antigen presenting cells (APCs), including DCs, LCs and macrophages, are strategically located to be initial target of the virus and to transfer infection to T lymphocytes (219, 237–239). We are just starting to have a more precise idea of the different APC subsets present in the genital mucosa and the specific role each of those subsets play in both HIV infection and anti-viral immune responses (230, 240–242). The distribution of immune cells as well as their ability to respond to a stimulus differs according to the female genital tract compartment and are distinct from systemic immune cells (243). The susceptibility of HIV target cells may vary depending on their localization at the mucosa and by their activation status. When compared to the endocervix or vagina, the ectocervix and the transformation zone have a larger concentration of intraepithelial CD4+ T cells and macrophages, indicating that this is likely where HIV transmission most frequently occurs (244). In addition, the CD4+ T cells from ectocervix appeared to be more sensitive to HIV infection compared to those from the endocervix and endometrium, probably because of their Th17 profile and high expression of CCR5 (245).

Collection of mucosal samples is often complicated by both technical and ethical reasons (246, 247) however, it is necessary to fill in the key knowledge gaps with respects to mucosal immunology. In addition, it would be relevant to collect paired vaginal and rectal tissues from women, as to directly compare the two mucosal sites.

## Closing the gender gap: Let’s give women access to a range of safe and efficacious PrEP including multi-purpose technologies (MPTs)

Women represent an important population to consider in the evolution of PrEP and unlike other HIV prevention strategies, a range of efficacious PrEP options create the opportunity to empower women to take control of their own health without requiring negotiation or consent from their partner. Yet, women are not always seen as a “key target” for HIV prevention and in many countries are not even considered as a group at risk of HIV infection (248). As a consequence, PrEP services focused mostly on key populations, such as MSM and, to a lesser extent, *trans*-gender women (248–251), and the overall utilization of PrEP by women remains low. In 2018 only 7% of woman were prescribed PrEP in the US (252) and the European Centre for Disease Prevention and Control in 2019 estimated that less than 10% of PrEP users in Europe were women (including *trans*-gender women) (253). Recent European survey studies evidenced that, while women’s access to PrEP remains limited (248), those who perceive themselves to be at high HIV risk are interested in using PrEP (254). Similar results were reported in two US survey studies within *cis-*women attending a family planning clinic or presenting for HIV/STD testing. Over 70-80% of participants reported not knowing PrEP was an option for them and over two-thirds declared they would probably or definitely like to take it (255, 256). According to a qualitative study that assessed PrEP acceptability among 30 *trans*-gender women in San Francisco, knowledge of PrEP was low, interest was relatively high and the opportunity to acquire PrEP from a *trans*-competent practitioner was reported as crucial for PrEP adoption and adherence (257).

Even though the majority of PrEP trials are being undertaken in *cis*-women in Africa, this is no guarantee for access or affordability. Inequities in access to HIV prevention and treatment for women remain a global challenge. Efforts should be put in increasing awareness among vulnerable women of the existence of PrEP as a viable preventive measure. In addition, health care professionals, researchers and community representatives should be involved in initiatives aiming to promote equality of access to care and implement PrEP availability for women across the globe; these initiatives should include a close dialogue with pharma companies involved in the design and production of PrEP drugs aimed at speed-up the development of HIV preventive measures tailored for women genital tract. Additional efforts are needed to make PrEP a successful prevention tool not only for men but also for women of all ages.

## Additional approaches to HIV prevention

Intersections between HIV and other sexually transmitted infections represent another potential avenue for HIV prevention interventions. For example, human papillomavirus (HPV) vaccination could decrease genital inflammation and HIV risk considering the established relationship between HPV infection, genital cytokines and increased HIV risk (258, 259). Similarly, considering the universal sub-optimal nature of syndromic management of STIs, opportunities exist to design appropriate point-of-care strategies to treat current infections and limit the development of a genital immune and microbial environment susceptible to HIV infection (260, 261). An important fact to consider is a possible increase in STIs prevalence following the distribution of long-acting ARVs or future HIV vaccines. Some evidence for this exists in MSM populations following oral PrEP (262), and, more recently, the resurgence of syphilis during the global COVID pandemic (263). Although the latter may in part be due to limitations to service delivery, additional efforts to avoid complacency and reductions in condom use or other risk-reducing efforts may need to be considered to prevent STIs on administration of interventions like HIV vaccination or long-acting ARV. It remains essential for research to focus on developing multipurpose interventions that women can use to prevent a range of viral and bacterial STIs, including HIV, and manage fertility simultaneously.

Given the limitations of available biomedical options for HIV-1 prevention in women, there is a critical need for new technologies. The use of broadly neutralizing antibodies (bNAbs) to HIV may represent an alternate strategy to overcome challenges seen with other available PrEP strategies. Compared with ART, PrEP mediated by bNAb can neutralize the free virus before the infection and act earlier than antivirals, with a reduced side effects profile expected and less concerns for the development of resistance when used in double or triple combinations. bNAbs have been engineered with the LS (lysine-serine) mutations to have a longer half-life *in vivo* (264). These modifications translate to less frequent administration compared to current PrEP strategies that require daily or intermittent administration. bNAbs can provide protection for a longer period and circumvents the adherence required for daily pill-taking or vaginal applications. The administration of bNAbs would also be unrelated to sexual intercourse and the delivery method could minimize stigma. However, there are limitations in the use of bNAbs due to a problem of costs and administration. In addition, given the global diversity of HIV-1, combination of bNAbs or multiple-specific antibodies will probably be needed to generate the essential breadth for efficient protection.

## Conclusions and future directions

Women and men have different biology, different sex organs, different hormones, different susceptibility to infections and different cultural influences, all of which can lead to difference in health and in the response to medical treatments. There is growing recognition that proper evaluation of important biological factors, such as sex and gender, is necessary to ensure the quality and generalizability of biomedical research. However, *cis-* and *trans*-gender women have historically been underrepresented in clinical trials, including but not limited to those evaluating HIV prevention strategies, although we acknowledge that over the past few decades, there has been a steady rise in their presence, partly as a result of laws, policies, regulations and guidance (265).

The underrepresentation of women in HIV clinical research has generated important knowledge gaps applying to both *cis*- and *trans*-women worldwide, from US to Europe to Sub-Saharan Africa. In order to fill these gaps, we should ensure that HIV research addresses specific sex and gender differences and supports the full participation and meaningful involvement of women across their life-course. Women participation in clinical trials and independent analysis of data for men and women are crucial to gain more knowledge about the biological differences between the sexes in HIV infection and to design sex-specific strategies to HIV treatment. Acknowledging these differences when planning and administering care can help address disparities. It takes dedication from basic, clinical, and social scientists, as well as education of women about the goals and importance of clinical research. Without it, putting a stop to the HIV epidemic will not be possible.

Including women in research is not just a matter of enrolling women in clinical trials, but also of involving female scientists and potential users in the conceptualization, design and implementation of these studies. Gender inequalities in science, technology, engineering, and mathematics (STEM) have been well documented (266–269). There is quite a lot of evidence that women in science need to have support networks to enhance their visibility, efficacy and bring unique skills to the scientific field. Efforts should be made to provide equal opportunities and encourage the participation of female scientists in the establishment of clinical cohorts and leading of clinical studies. Sustainable development and the contribution that women make to science and technology are inextricably linked; neither can exist apart.

## Author contributions

MC wrote the initial draft. All authors revised the manuscript and approved the submitted version.
